# 3.0-T MR-guided transgluteal in-bore-targeted prostate biopsy under local anesthesia in patients without rectal access: a single-institute experience and review of literature

**DOI:** 10.1007/s00261-024-04183-1

**Published:** 2024-02-21

**Authors:** Kaustav Bera, Nikhil Ramaiya, Raj Mohan Paspulati, Dean Nakamoto, Sree Harsha Tirumani

**Affiliations:** 1 Department of Radiology, University Hospitals Cleveland Medical Center, Cleveland, OH 44106 USA; 2 Department of Diagnostic Imaging and Interventional Radiology, Moffitt Cancer Center, Tampa, FL 33612 USA; 3 Department of Radiology, Louis Stokes Cleveland VA Medical Center, Cleveland, OH 44106 USA

**Keywords:** Local anesthesia, MR guidance, Prostate biopsy, Prostate cancer, Trasngluteal in-bore-targeted biopsy

## Abstract

**Purpose:**

To describe the technique and evaluate the performance of MRI-guided transgluteal in-bore-targeted biopsy of the prostate gland under local anesthesia in patients without rectal access.

**Methods:**

Ten men (mean age, 69 (range 57–86) years) without rectal access underwent 13 MRI-guided transgluteal in-bore-targeted biopsy of the prostate gland under local anesthesia. All patients underwent mp-MRI at our institute prior to biopsy. Three patients had prior US-guided transperineal biopsy which was unsuccessful in one, negative in one, and yielded GG1 (GS6) PCa in one. Procedure time, complications, histopathology result, and subsequent management were recorded.

**Results:**

Median interval between rectal surgery and presentation with elevated PSA was 12.5 years (interquartile range (IQR) 25–75, 8–36.5 years). Mean PSA was 11.9 (range, 4.8 -59.0) ng/ml and PSA density was 0.49 (0.05 -3.2) ng/ml/ml. Distribution of PI-RADS v2.0/2.1 scores of the targeted lesions were PI-RADS 5–3; PI-RADS 4–6; and PI-RADS 3–1. Mean lesion size was 1.5 cm (range, 1.0–3.6 cm). Median interval between MRI and biopsy was 5.5 months (IQR 25–75, 1.5–9 months). Mean procedure time was 47.4 min (range, 29–80 min) and the number of cores varied between 3 and 5. Of the 13 biopsies, 4 yielded clinically significant prostate cancer (csPca), with a Gleason score ≥ 7, 1 yielded insignificant prostate cancer (Gleason score = 6), 7 yielded benign prostatic tissue, and one was technically unsuccessful. 3/13 biopsies were repeat biopsies which detected csPCa in 2 out of the 3 patients. None of the patients had biopsy-related complication. Biopsy result changed management to radiation therapy with ADT in 2 patients with the rest on active surveillance.

**Conclusion:**

MRI-guided transgluteal in-bore-targeted biopsy of the prostate gland under local anesthesia is feasible in patients without rectal access.

## Introduction

In the USA, prostate cancer is the most common cancer among men with about 288,300 new cases every year with about 1 in 8 men being affected by prostate cancer during their life time [1]. While Prostate-specific antigen (PSA) is used as a screening tool [2], multiparametric MRI (mp-MRI), is widely used for prostate cancer diagnosis and detection [3, 4]. Several organizations now recommend mp-MRI in biopsy naïve patients who present with increased PSA [5]. Prostate biopsy is the gold standard for diagnosis of prostate cancer. Systematic transrectal ultrasound (TRUS)-guided biopsy is the most common technique for prostate biopsy [6]. Limitations of this technique include overdiagnosis of clinically insignificant cancers besides also having a high rate of false-negative biopsies. Hence, there has been increasing use of mp-MRI and MRI-directed biopsies [7, 8]. MRI-directed biopsies are performed either by fusing the MRI images with ultrasound through transrectal or transperineal route (MR-TRUS fusion biopsies) [9] or in-bore transrectal route under direct visualization within the MRI scanner. These techniques require access through the rectum for needle guidance. In a few institutions, in-bore transperineal prostate biopsy is performed under direct MRI visualization. However, this technique is not widely available.

Patients with prior surgery on rectum in the form of proctocolectomy for inflammatory bowel disease (IBD) or abdominoperineal resection (APR) for rectal cancer lack rectal access and therefore pose a diagnostic challenge when they present with elevated PSA. With increasing life expectancy of both IBD and rectal cancer patients, presentation with elevated PSA in this cohort may not be uncommon. Although there is no clear correlation between IBD and prostate cancer, it has been shown that men with UC ulcerative colitis have a higher risk of developing prostate cancer [10]. While some IBD patients with ileal pouch-anal anastomosis can get transperineal biopsy with ultrasound guidance through the ileal pouch [11, 12], it may not always be feasible owing to strictures at the anastomosis. Ultrasound-guided or in-bore transperineal prostate biopsy is an option in this setting but can be challenging due to complexity of the procedure, excessive fibrosis, and limited availability as well as requirement of general anesthesia in case of in-bore transperineal biopsy.

Accordingly, the purpose of this study is to describe the technique and evaluate the performance of MRI-guided transgluteal in-bore-targeted biopsy of the prostate gland under local anesthesia in patients without rectal access.

## Methods

### Study population

This HIPAA compliant retrospective study was approved by the institutional review board with waiver for informed consent. We searched the radiology database at our tertiary care hospital between January, 2016 and August, 2023 to identify patients without rectal access who underwent MRI-guided transgluteal in-bore-targeted biopsy of the prostate gland. The search returned 13 procedures in 10 men (mean age, 69 (range 57–86) years), with three patients undergoing a repeat biopsy. Clinical information (Table 1) was extracted from the electronic medical records including management after the biopsy and the date of last follow-up.

**Table 1:** Patient characteristics as well as procedural details of *n* = 10 patients

	Patient 1	Patient 2	Patient 3	Patient 4	Patient 5	Patient 6	Patient7	Patient 8	Patient 9	Patient 10
Age (y)	78	65	67	74	57	70	86	62	62	65
Race	White	White	White	White	White	White	White	White	White	White
PSA (ng/ml)	8.2	5.7	6.4	59	4.8	8	5.7	5.3	7.6	8.6
PSAD (ng/ml/ml)	0.16	0.17	0.13	3.2	0.05	0.26	0.13	0.1	0.4	0.3
Time between MRI and biopsy (in months)	1	2 (first) 11 (second)	8 (first) 10 (second)	1	12	1	1 (first) 8 (second)	5	6	3
No of biopsy cores obtained	3	4 (first) 4 (second)	4 (first) 4 (second)	4	4	3	3 (first) 3 (second)	3	5	4
Tissue yield	NA	NA (first) Benign, Single atypical, Benign, and 5% in four cores, respectively	NA (first) 95%, 80%, 70%, and 60% in four cores	99% in all four samples	NA	60%, 35% in 2 cores Benign 3rd core	NA NA	NA	NA	30%, Benign, 35%, 25%
ISUP GG from transgluteal MRGB (maximum across lesions)	Benign	Benign (first) Group 2; 3 + 4 = 7 in one core (second)	Benign (first) Group 2; 3 + 4 = 7 (second)	Group 4; 4 + 4 = 8	Benign	Group 1 (3 + 3 = 6) from Left PZ	Benign Left (first) Benign right (second)	Benign	Benign	Group 2 (3 + 4 = 7) from 2 cores; Group 1 (3 + 3 = 6) from 1 core
PI-RADS v2 scores	4—Left PZ	4—Right PZ	5—Left PZ	5—Entire PZ	4 – Left PZ	4- Left PZ	4 – Left PZ	3—Right PZ	4—Left TZ	5—Left PZ
Size of the biopsied lesion on MRI	1.4 cm	1.0 cm	1.5 cm	3.6 cm	1.0 cm	1.0 cm	1.0 cm	1.0 cm	1.3 cm	2.1 cm
Average procedure time	35 min	43 min (1st) 33 min (2nd)	70 min (1st) 48 min (2nd)	80 min	36 min	53 min	38 min (1st) 69 min (2nd)	40 min	42 min	29 min
Duration of follow-up (Date of biopsy – Date of last office visit)	12 months	0.5 month	10 months	25 months	32 months	34 months	44 months	2 months	0.5 month	1 month
Time to PSA Increase from prior rectal surgery	10 years	6 years	35 years	7 years	10 years	39 years	38 years	9 years	6 years	15 years
Previous prostate biopsy status	US guided—Negative	None	US guided—ISUP GG 1 (3 + 3 = 6)	None	US guided—Attempted but unsuccessful	None	None	None	None	None
Reason for proctectomy	UC	UC	UC	Rectal cancer	UC	UC	UC	UC	Rectal cancer	Rectal cancer
Type of bowel surgery	Total proctocolectomy and ileostomy	Total proctocolectomy and ileostomy	Total proctocolectomy and ileostomy	Abdominopelvic resection (Partial colectomy, proctectomy, colostomy)	Total proctocolectomy with J-pouch	Total proctocolectomy and K-pouch	Total proctocolectomy and end ileostomy	Total proctocolectomy and end ileostomy	Abdominopelvic resection (Partial colectomy, proctectomy, colostomy)	Neoadjuvant chemoradiaton; Abdominopelvic resection (Partial colectomy, proctectomy, colostomy) and excision of posterior wall of prostate capsule followed by post-operative radiation
Current therapy for PCa	Active surveillance	Active surveillance	Radiation and ADT	Radiation and ADT	Active surveillance	Active surveillance	Active surveillance	Active surveillance	Active surveillance	Active surveillance

Rectal access was not possible due to prior total proctocolectomy with end ileostomy or J-pouch or K-pouch for ulcerative colitis in 7 patients and prior abdominoperineal resection for rectal cancer in 3 patients. Three patients in the cohort had prior US-guided transperineal biopsy which was unsuccessful in one, negative in one, and yielded GG1 (GS6) PCa in one. All patients underwent pre-biopsy PI-RADS compliant MRI at our institute. The scans were reviewed retrospectively by two radiologists to assign PI-RADS V2.1 scores.

### MRI-guided transgluteal in-bore-targeted biopsy technique

A routine urine culture was performed within two weeks before the biopsy and any anticoagulation was withheld 5 days before the procedure unless there was a contraindication. Patients continued 81-mg aspirin if clinically indicated. Routine laboratory work-up was performed to exclude coagulopathy. Prophylactic intramuscular antibiotic injection was given on the day of the procedure.

**Fig. 1:**
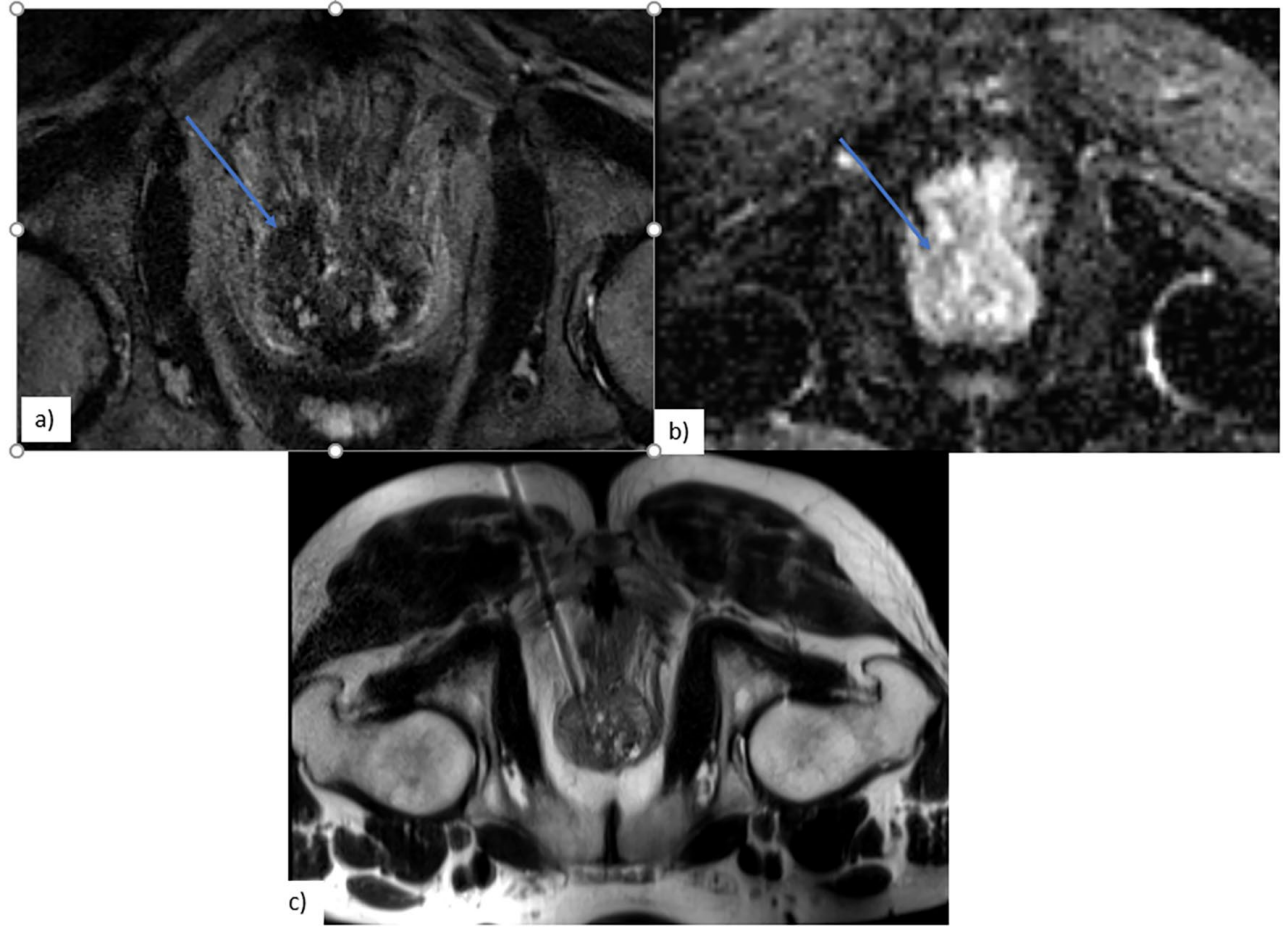
67-year-old man with elevated PSA (6.4 ng/ml). Blue arrow in Intra-procedural Axial T2 **a**. ADC **b** demonstrates a 1.5-cm PI-RADS 5 lesion in the left peripheral zone. Intra-procedural T2 fast spin-echo **c** demonstrates the needle in target lesion. Histopathology revealed a Gleason Group 2 (3 + 4 = 7) lesion in all four cores (95%, 80%, 70%, and 60% yield in four cores, respectively)

All in-gantry biopsies were performed on a 3.0-T MRI scanner (Verio; Siemens Healthineers). The patient was placed prone and head first on the MR table. MR-compatible skin markers were placed on the skin of the buttocks on the side of the lesion in the prostate gland. After obtaining axial T2 fast spin-echo images covering the prostate anatomy and in some cases of diffusion-weighted images (b = 0, 500, 1000, 1400), the focal lesion in the prostate gland was localized (Fig. 1). The skin entry site was marked according to the shortest distance to the region of interest (ROI) in the prostate gland through the gluteal muscles and periprostatic fat. The biopsy site was then prepped and draped followed by local anesthetic infiltration. Conscious sedation was used in only one procedure. MRI compatible 16/17-gauge coaxial needle was advanced through the gluteal muscles obtaining serial axial T2 fast spin-echo images to monitor the advancement of the needle (Fig. 1). Care was taken to negotiate fibrous tissue and avoid bowel loops in case of ileal J-pouch anastomosis without excessively deviating from the planned needle trajectory. Once the coaxial needle tip was in a satisfactory position on the prostate capsule in the vicinity of the ROI, MR-compatible 18-gauge semiautomated spring loaded biopsy gun with a cutting needle that has 2 cm throw was advanced through the introducer and deployed in the prostate gland ROI. Tissue sample was obtained after confirming the position of the biopsy needle in the ROI. Additional samples were obtained by making minor changes in the angle of the coaxial and biopsy needles to avoid sampling errors due to blood clots in biopsy tract. Post-procedure scans were obtained after removing the biopsy and introducer needles. Patient was observed for 2 h before discharge from the hospital.

#### Data analysis

Placement of the needle in the prostate gland and obtaining prostate tissue in the core biopsy sample was deemed as technical success. The procedure time was calculated from time stamps on the planning scan and the post-procedure check scans. The procedure notes were reviewed to obtain information about number of core samples and the post-procedural complications. The electronic medical records were reviewed for office or emergency visits which were attributable to the procedure. The histopathology result and the management after the biopsy were also recorded. The 2014 International Society of Urology Pathology Grade Group (IUSP-GG) classification system was utilized to describe the prostate biopsy results [13]. IUSP-GG 0 was considered no cancer, IUSP-GG 1 (Gleason score = 6) considered low-grade or indolent prostate cancer, and IUSP-GG ≥ 2 (Gleason score ≥ 3 + 4) was considered clinically significant prostate cancer (csPCa).

## Results

The median interval between rectal surgery and presentation with elevated PSA was 12.5 years (interquartile range (IQR) 25–75, 8–36.5 years). The mean PSA was 11.9 (range, 4.8 -59.0) ng/ml and PSA density was 0.49 (0.05 -3.2) ng/ml/ml. The distribution of PI-RADS v2.0/2.1 scores of the target lesions were PI-RADS 5–3; PI-RADS 4–6; and PI-RADS 3–1. The mean size of the target lesion was 1.5 cm (range, 1.0–3.6 cm). The median interval between the mp-MRI and the biopsy was 5.5 months (IQR 25–75, 1.5–9 months). The average procedure time was 47.4 min (range, 29–80 min). The number of core biopsies varied between 3 and 5 per procedure. The mean skin to target distance was 10.9 cm (range, 8.6–14.5 cm).

Twelve of the thirteen biopsies (92%) were technically successful with prostatic tissue present at histopathology. Out of the 13 biopsies, four (31%) yielded clinically significant prostate cancer (Gleason score ≥ 7), one yielded insignificant prostate cancer (Gleason score = 6), seven yielded benign prostatic tissue, and one did not have prostatic tissue (unsuccessful biopsy). Overall, the cancer detection rate was 5 out of 13 biopsies (38.5%). The transgluteal in-bore biopsy was repeated in 3 patients. The biopsy was repeated due to rising PSA and benign tissue on initial biopsy in two patients with PI-RADS 4 and 5 lesions, respectively, on MRI. One of them had upgrading of the cancer on repeat biopsy and the other had benign tissue on repeat biopsy. The third patient had repeat biopsy due to initial unsuccessful biopsy and PI-RADS 4 lesion on MRI with clinically significant cancer on repeat biopsy. Please see Table 1 for complete description.

All the biopsies were well tolerated and did not lead to any immediate major or minor post-operative complications. One patient, while not experiencing any immediate post-operative complications presented to the emergency four days later and was found to have obstructive ureteral calculus.

The median duration of follow-up after the biopsy was 11 months (IQR 25–25, 1–32 months). The management after the biopsy was changed to radiation therapy with ADT in 2 patients with csPCa on biopsy. The rest of the patients continued active surveillance at the time of last follow-up.

## Discussion

In this study, we have described a transgluteal approach to perform 3.0-T MR-guided targeted prostate biopsy under local anesthesia in a cohort of patients without rectal access. Conscious sedation was used in only one procedure. The technical success was 92% with cancer detection rate of 38.5% and 31% yield of csPca. The procedure was tolerated well with no major or minor immediate post-procedure complications.

Transgluteal in-bore-targeted prostate biopsy has been reported previously [14–16]. Zangos et al. [15] published a feasibility study in an open low-field MRI with 25 patients using a transgluteal in-bore approach with a 40% (10/25 patients) yield of carcinoma, similar to our study. Bodelle et al. [14] performed 1.5-T MRI in-bore transgluteal prostate biopsies in 25 men with cancer detection rate of 35%. Fischbach et al. [16] reported a cancer detection rate of 63% in 30 patients with 3.0-T MR in-bore transgluteal-targeted prostate biopsy. The procedure time was 11 min, 31 ± 7 min, and 26 min, respectively, in these studies compared to 47 min in our study. These studies did not report history of prior ano-rectal surgery in their patients in contrast to our cohort which can explain the relatively longer procedure time in our study.

Few studies have reported MR-guided in-bore biopsy using the transperineal approach. Two such studies from the same group used a needle guide template with the patient in lithotomy position and had a cancer detection rate of slightly over 50% and a procedure time of slightly less than 2 hours [17, 18]. However, in-bore transperineal technique is not widely available and requires general anesthesia in contrast to local anesthesia in our study with comparable cancer detection rate (38.5%). Ultrasound-guided transperineal prostate biopsy using only local anesthesia has been reported previously but in patients with intact rectum and no prior pelvic surgery [19, 20].

Prostate biopsy either transperineal or transgluteal without rectal access can be challenging due to change in pelvic anatomy and post-surgical fibrosis. Previous studies in this subset of patients used either US [21] or CT guidance [22–27] for prostate biopsy. In the study by Hansen et al. [21], ultrasound-guided transperineal biopsy with cognitive registration of MR images was successful in 9 out of 11 patients and yielded cancer detection rate of 78%. The biopsies in this study were performed by an urologist under the transperineal ultrasound guidance of experienced radiologist. While transperineal us guidance for prostate biopsy is safe in experienced hands, it can be challenging due to poor definition of the prostate gland especially when there is post-surgical fibrosis. Presence of small bowel loops in patients with ileal pouch can result in poor acoustic window for US guidance. Insertion of transpouch US probe can be challenging in patient with anastomotic stricture. Transperineal biopsy with transrectal US guidance was unsuccessful in one of three patients prior to MR-guided in-bore biopsy in our study due to the inability to advance the ultrasound probe across the staple line. MR-guided transgluteal in-bore biopsy can overcome these limitations by providing direct visualization of the needle path to help avoid bowel or bladder injury.

CT-guided transgluteal prostate biopsy with random sampling was evaluated in several studies with technical success of ≥ 95% and cancer detection rate of 40–60% [23–27]. The procedures in these studies were performed under local anesthesia with conscious sedation in some and required more than one site of percutaneous access for systematic sampling of the gland. However, only random prostate biopsies can be performed under CT. Targeted biopsy of focal lesions cannot be performed with CT guidance since focal lesions cannot be differentiated from normal prostate with CT. The ability of MRI to clearly demonstrate the focal lesion along with real-time visualization of the needle tip in the target lesion offers a clear advantage over CT guidance. Repeat transgluteal biopsy was performed in three patients in our cohort with csPCa detected in two out of the three patients. Similar to prior studies [25, 26], this supports the need for repeat biopsy after initial negative biopsy.

MR in-bore transgluteal biopsy was safe in our study with no major or minor complications in any of the patients. This is similar to most of the prior studies. Minor complications were reported in the study by Bodelle et al. [14] with transgluteal MR in-bore biopsy. With CT-guided biopsies, minor complications in the form of hematuria and periprostatic hematoma were reported in the studies by Goenka et al. [26] and Olson et al. [27], respectively.

There are inherent limitations to our retrospective study of small sample size from a single institute and therefore, the results of our study may not be applicable to other practices. We also do not have follow-up biopsies in patients with benign tissue on the initial biopsy except for two patients. Lack of comparison arm with another technique like transperineal approach is also a limitation. However, such comparative studies in this select group of patients may be possible in multi-institutional studies in future.

In conclusion, 3.0-T MRI in-bore transgluteal prostate biopsy is a safe technique in patients who do not have rectal access and can be performed under local anesthesia.
